# Urrets-Zavalia syndrome following implantable collamer lens (ICL) implantation: a case report and review of the literature

**DOI:** 10.1186/s13256-025-05255-6

**Published:** 2025-05-12

**Authors:** Ke Li, Ting Huang, Juan Yuan, Siyi Bao

**Affiliations:** Department of Ophthalmology, Daping Hospital, Army Medical Center of PLA, Army Medical University, No.10, Changjiang Branch, Daping, Yuzhong District, Chongqing, 400042 China

**Keywords:** Urrets-Zavalia syndrome, Implantable collamer lens implantation, Dilated pupil, Vault, Intraocular pressure

## Abstract

**Background:**

Urrets-Zavalia syndrome is a condition that arises after eye surgery, often linked to increased intraocular pressure following the procedure. We present a case of a progressively dilated and fixed pupil in one eye following the implantation of a toric implantable collamer lens. There are no documented cases in the literature regarding this condition in China. The patient gradually and completely recovered after our intervention. This case differs from all previous implantable collamer lens implantation cases, and the treatment method used is unprecedented.

**Case presentation:**

A 33-year-old Han Chinese woman successfully had a toric implantable collamer lens implanted in both eyes. The left eye surgery was performed on the first day. Within 10 days post-operation, the pupil of the left eye continued to dilate. We considered the cause of pupil dilation in this case to be pupillary sphincter paralysis caused by elevated intraocular pressure and the excessive size of the toric implantable collamer lens. Therefore, the toric implantable collamer lens needed to be replaced with a smaller one. However, it would take 3–4 months to prepare the new toric implantable collamer lens. After discussing the situation with the patient and obtaining her consent, we repositioned the toric implantable collamer lens to the new target location. The alternative toric implantable collamer lens was ultimately available more than 2 months later. After the replacement operation, the pupil gradually returned to normal.

**Conclusion:**

The vault is not the only criterion for determining whether the size of an implantable collamer lens is appropriate. In this case, it can be concluded that the potential reason for pupil dilation was that the size of the toric implantable collamer lens was too large, and it reversed after changing to a smaller size. The dilated pupil would not return to normal if the toric implantable collamer lens were not replaced. When persistent mydriasis (Urrets-Zavalia syndrome) occurs after implantable collamer lens implantation, it is essential to determine whether the implantable collamer lens size is appropriate besides controlling the intraocular pressure. In addition, alternative toric implantable collamer lenses often need to be customized. During long waiting times, provisional implantable collamer lens realignment will predictive simulation for planned exchange.

## Introduction

Urrets-Zavalia syndrome (UZS), characterized by a fixed dilated pupil, was first reported by Alberto Urrets-Zavalia in 1963. He described six cases of atrophic and mydriatic pupils following penetrating keratoplasty (PKP) [1]. UZS has also been observed in cataract surgery, glaucoma surgery, and intraocular lens implantation in phakic eyes [2–6]. It is generally agreed that this syndrome is related to high intraocular pressure (IOP) and iris ischemia.

Implantable collamer lens (ICL) implantation is one of the most common myopia correction surgeries. The total number of such surgeries has risen rapidly in recent years. The incidence of complications following ICL, including cataract, high intraocular pressure, and unexpected visual acuity, is low [7, 8]. There are also several cases of mydriasis after ICL implantation [6, 9]. However, relevant cases have not been reported in the formal literature from China. Most patients with dilated pupils after ophthalmic surgery cannot fully recover, which can cause irreversible damage to patients. Complications can significantly impact patient outcomes following ICL implantation and may also affect their quality of life.

In this case, progressively dilated and fixed pupil occurred in one eye after toric ICL (TICL) implantation. After determining the patient’s condition, the intervention started approximately 2 weeks after the implantation. The patient completely recovered after the intervention. The whole process of this case is complicated and involves many difficulties. Therefore, this experience can be shared as a reference for other ICL surgeons.

## Case report

**Fig. 1:**
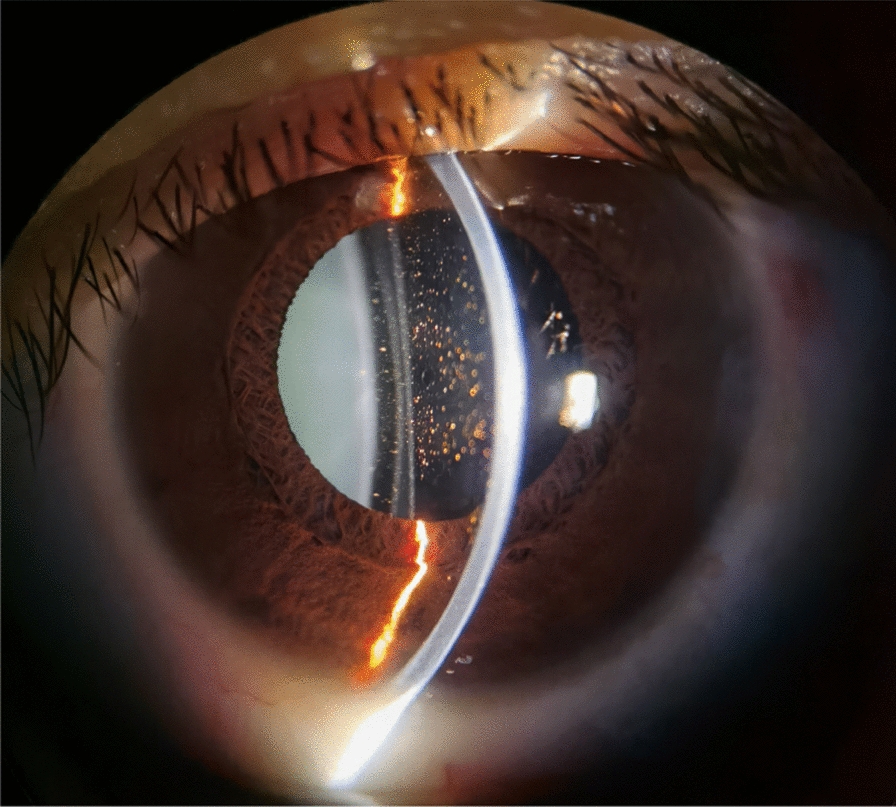
One day after the left eye surgery, the pupil dilated and fixed, with more pigments in the anterior chamber

**Fig. 2:**
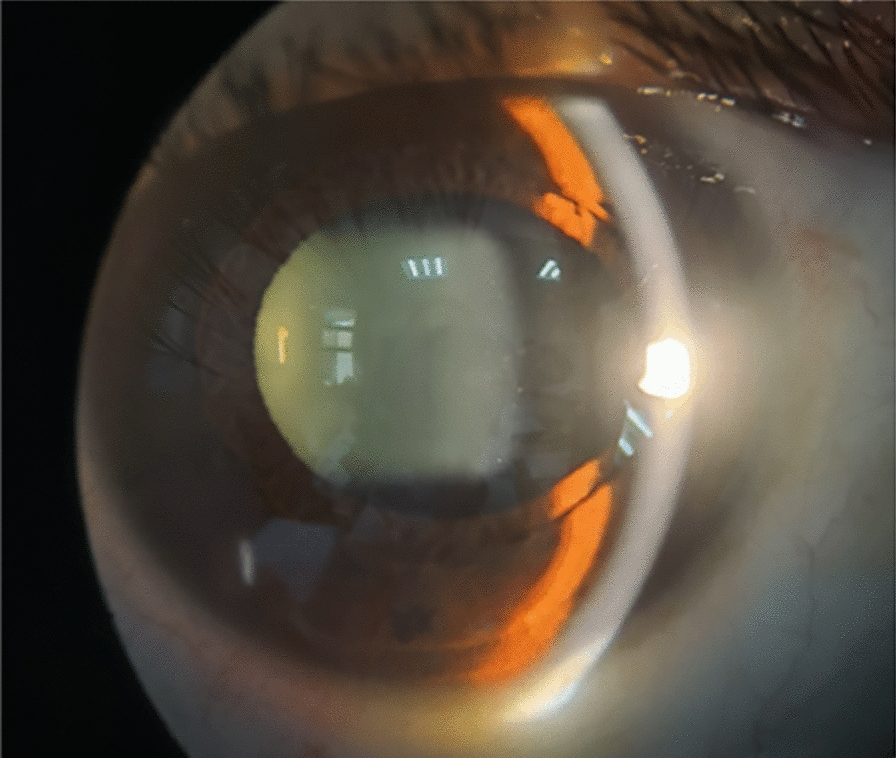
Six days after the left eye surgery, the pupil further dilated and fixed

**Fig. 3:**
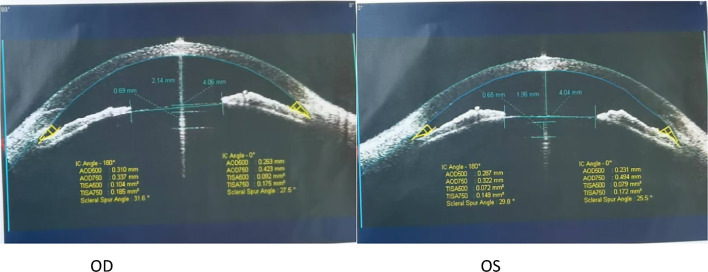
Postoperative vault in both eyes 1 week after surgery (measured by anterior segment optical coherence tomography, Zeiss); pilocarpine eye drops were applied in the left eye

**Fig. 4:**
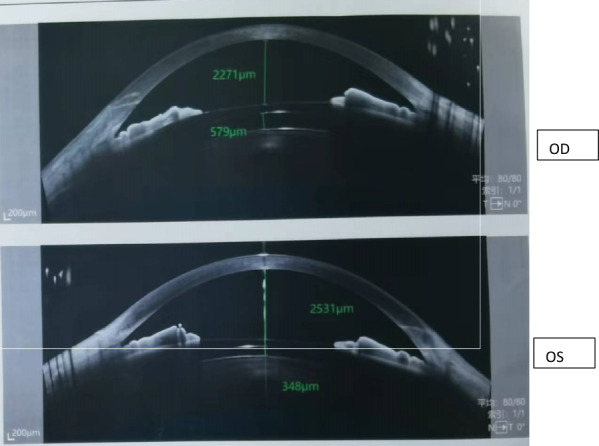
Postoperative vault in both eyes 1 month after the replacement surgery (measured by anterior segment optical coherence tomography, TowardPi, Beijing)

**Fig. 5:**
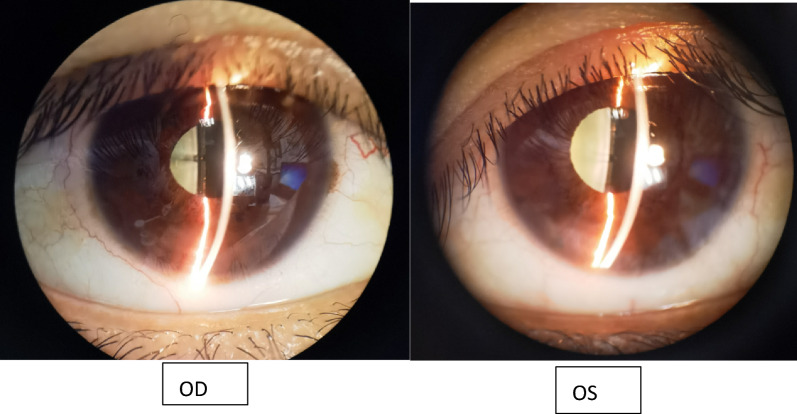
Slit-lamp examination of left eye 3 months after replacement surgery

A 33-year-old woman with no significant medical history successfully underwent TICL (V4c TICL, STAAR Surgical, Switzerland) implantation in both eyes on separate days to correct her bilateral myopia with astigmatism. Preoperative best-corrected visual acuity (BCVA) was 20/20 in the right eye (OD) with −4.75 −1.25 × 180° and 16/20 in the left eye (OS) with −5.50 −2.25 × 5°. The TICL rotates 17° clockwise after horizontal implantation in the left eye. The left eye surgery was performed on the first day. When the patient left the hospital, the cornea was transparent, the anterior chamber reaction was mild, the aqueous humor was clear, and the IOP was 15 mmHg. On the morning of the second day, she felt slightly dizzy and bloated. However, it did not attract her attention owing to her good vision. Surgery on the second eye was scheduled for the following afternoon, and the first eye was reviewed at 1 p.m. According to the results of the reexamination of the left eye, the visual acuity was 20/20, and the IOP was 40 mmHg. Slit lamp examination showed that the cornea was transparent. Many pigments floated in the anterior chamber. The pupil was dilated to approximately 4–5 mm, and the pupillary light reaction was absent (Fig. 1). The patient received IOP-lowering and anti-inflammatory treatment. The right eye was implanted with a TICL on the second day because the patient insisted on continuing the surgery. The sizes of the TICLs in both eyes were the same. She left the hospital after a 4-h postoperative observation. Her IOP was 20 mmHg in the right eye and 26 mmHg in the left eye when she left. Postoperative examinations were performed on the third day. The vision acuity of both eyes was 20/20. The IOP of the right eye was 15 mmHg, and the IOP of the left eye was 18 mmHg. The IOP of both eyes remained normal since then. The cornea of the left eye was transparent. The anterior chamber pigments were significantly reduced. The size of the pupil in the left eye was still 4.5 mm, and no pupillary light reflex was observed. The patient was instructed to take routine medication and was re-examined around a week after the operation. Six days later, the patient returned to the hospital because the pupil of her left eye had further enlarged. The slit-lamp examination showed that the pupil of her left eye had dilated to approximately 6 mm, appearing slightly elliptical in shape, with no light reflex (Fig. 2). The vault volumes of both eyes were normal, approximately 1 cornea thickness (CT) (vault: the distance from the anterior surface of the lens to the posterior surface of the ICL). According to anterior segment optical coherence tomography (AS-OCT), the vault was 690 µm in the right eye and 650 µm in the left eye (Fig. 3). The left eye’s measurement was taken after pupil contraction. The pupil of the left eye could contract to approximately 4 mm after applying pilocarpine eye drops. The vault of the left eye was approximately 1.2 CT after the contraction of the pupil. The patient was subsequently administered pilocarpine eye drops 3 times per day. However, on the ninth day post-operation, the examination indicated that the pupil of the left eye appeared more dilated. Slit-lamp examination revealed that the pupil of the left eye was fixed at approximately 7 mm. After four drops of pilocarpine, the pupil could constrict to a certain extent but was not smaller than 4.5 mm. On the 12th day after the operation, the vault in the left eye was measured by AS-OCT before the pupil constriction and was found to be 390 µm. During the examination, the oval-shaped dilation of the pupil was somewhat consistent with the placement of the TICL lens. In addition, the vault of the left eye increased after pupil contraction instead (contrary to common practice). Thus, it is suspected that the pupil dilation was linked to the oversized TICL, despite the vault being considered “normal.” The TICL needed to be replaced with a smaller one. The size of the original TICL was 12.6 mm horizontally and rotated 17° clockwise. The alternative smaller TICL was 12.1 mm or 12.6 mm vertically. The toric ICL should not be too small in case the vault becomes too low and the astigmatic position may be unstable after implantation. Therefore, a 12.6-mm vertical TICL was considered the most appropriate size. However, the 12.6 mm vertical /12.1 mm TICL needed to be prepared for 3–4 months owing to the COVID-19 epidemic. Moreover, the effect of 12.6 vertical/12.1 TICL replacement or even ICL removal was unclear. After fully communicating with the patient and obtaining the patient’s consent, the TICL lens in the patient’s left eye was realigned to the vertical position from the original position. There was no additional mydriasis used before the realignment surgery. In this way, it was possible to know in advance whether the size of the alternative TICL was appropriate. While the postoperative visual acuity of the left eye was 20/50, it did not bother the patient much as the left eye is non-dominant and the patient was longing for the recovery of the pupil. The pupil of the left eye did not dilate further after the realignment operation and gradually decreased and became round. A slight light reflex was observed in the left eye 2 weeks after the realignment operation. The pupillary light reflex of the left eye was normal around 1 month after the realignment operation. This indicates that the treatment was effective, so the replacement of TICL would be implemented. One month after the realignment surgery, an ultrasound biomicroscopy (UBM) examination was performed, and several small cysts were found at 3–5 o’clock in the left eye, which was not shown by the UBM before implantation. However, no further examination was conducted as it was unclear whether it was related to this case. The alternative TICL became available more than 2 months after the first operation. On the 75th day after the first implantation, the left eye underwent TICL replacement surgery, which changed the TICL from 12.6 mm horizontally to 12.6 mm vertically. The degree of rotation of the intraocular lens was 4° clockwise in the vertical orientation. The pupil dilated only once before the operation. Carbachol injection was used to constrict the pupil immediately after replacement surgery. After that operation, the left eye pupil gradually returned to normal. One month after the replacement surgery, the pupil measured approximately 5–6 mm in diameter, and the light reflex was normal. The vaults of both eyes were normal (Fig. 4). Three months after the operation, the pupil completely recovered, and the light reflex was normal (Fig. 5). The visual acuity of both eyes was 20/20. The IOP and vault were both normal.

## Discussion

The exact pathogenesis of UZS is unclear and may be multifactorial. The most accepted theory is iris ischemia, along with a sudden increase in intraocular pressure [10, 11]. It has also been reported that UZS could be a complication of toxic anterior segment syndrome [12]. The risk factors related to UZS reported in literature include increased IOP, administration of a mydriatic agent, keratoconus, residual viscoelastics, and inflammation in the anterior chamber during or after surgery [13]. These risk factors are present in nearly every ICL implantation, with the exception of keratoconus. Furthermore, reports indicate that certain noninvasive procedures, including laser myopia correction surgery and lamellar keratoplasty, may also lead to postoperative UZS [14, 15].

The prognosis of patients with UZS also varies. The pupil will partially recover if the IOP is quickly controlled and there is no continuous iris stimulation. If the IOP increases for a long duration, the pupils will not return to normal. Two cases of UZS after toric ICL implantation have been reported. In these patients, even with quick IOP control, the patient’s pupils did not fully recover [6, 9]. Al Habash et al. did not consider the possibility that the TICL size was too large because the postoperative vault appeared normal [6]. Fraenkel et al. [9] believed that the size of the TICL was too large after implantation. Hence, the TICLs of both eyes were replaced. A few weeks later, the patient’s pupils were still dilated and anterior subcapsular cataracts developed. Finally, the patient’s eyes underwent phacoemulsification and intraocular lens implantation, but the pupils did not recover. Pupil dilation and fixation were reported in a Chinese patient after intraocular lens implantation, and returned to normal 2 months postoperatively [16]. Unlike the ICL, which is a posterior chamber intraocular lens, an anterior chamber intraocular lens was implanted in that patient. The pupil should be contracted rather than dilated before the operation. An iridectomy was performed 1 week before the procedure. After the implantation, IOP elevation and corneal edema occurred. After controlling IOP, the stimulation disappeared, and the dilated pupil fully recovered as a result [16].

The case described in this article differs from all previous ICL implantation cases, and the treatment method used is unprecedented. The operation proceeded smoothly, accompanied by only a slight postoperative reaction. The patient could leave home only if the IOP was normal. After returning home, the IOP was only moderately elevated. The maximum IOP recorded was 40 mmHg, and there was no corneal edema. Her visual acuity remained normal. In addition, the IOP was quickly controlled. Within 10 days after controlling the IOP, the pupil continued to dilate and the vault remained within the normal range. Therefore, the pupil dilation in this case was attributed to pupillary sphincter paralysis resulting from elevated intraocular pressure and the large size of the TICL.

Previous studies have shown that the reaction to 2% pilocarpine can be used as a potential predictor of pupil recovery [17]. In our case, the effect of pilocarpine on pupil contraction was also observed. However, this effect gradually weakened, indicating that sphincter function was gradually decreased.

One month after the reposition operation, UBM revealed several small ciliary cysts in the ciliary sulcus at 3–5 o’clock in the left eye. Owing to the limitations of the device, the images were not clear enough. During the first implantation operation, the TICL rotated horizontally 17° in a clockwise direction. The friction between the ICL’s loops and cysts may result in partial loss of pigment. The pigment can block the chamber angle and cause a temporary increase in IOP, ultimately leading to iris ischemia and pupil dilation. After a certain degree of damage to the pupillary sphincter, the TICL appeared relatively large. Continuous pressure between the ICL and iris stimulated the ciliary body, resulting in progressive mydriasis. Generally, an appropriate ICL lens is considered to have a normal vault between 250 and 750 μm (approximately 0.5–1.5 CT). Although the postoperative vault is the most important indicator for determining whether the size of the ICL is appropriate, surgeons should not focus only on the vault. In this patient, the vault was 1 CT after the initial implantation. After repositioning to a vertical position, the vault was still normal at approximately 0.8 CT (348 µm as measured by AS-OCT). However, when the TICL was in the horizontal position, the vault increased after pupil constriction (which is not typical). When the TICL was in the vertical position, the vault decreased after pupil constriction (which is typical). It showed that the TICL was too large in the horizontal position, resulting in continuous contact between the ICL and the iris/ciliary body, and leading to constant damage to the sphincter and dilation of the pupil. The size of the ICL became appropriate when it was in the vertical position. Therefore, it is suggested that the vault should not be considered as the sole determinant for assessing ICL size. Even if the vault is proper, the ICL may be too large, causing continuous irritation to the ciliary body. Some surgeons may remove the TICL directly to observe the recovery of the pupil in similar cases. In contrast, if the patient’s pupil dilation cannot recover, whether to reimplant a new ICL and what type of ICL to reimplant will become challenging problems. Although the patient’s pupil dilated and she may experience photic phenomena, she still desires to maintain functional uncorrected visual acuity. The realignment operation is a more straightforward choice than replacing or removing the ICL. As such, if the cause of pupil dilation cannot be ultimately determined, surgeons can remove the ICL and establish further plans until the pupil is fully recovered, if possible.

## Conclusion

The vault is not the sole factor in determining the appropriate size of an ICL. In this case, the pupil dilated gradually even though the vault remained within normal limits post-implantation. It appears that pupil dilation was potentially caused by an oversized TICL, which may be resolved by replacing it with a smaller model. The dilated pupil remained abnormal until the TICL was replaced. When persistent mydriasis (UZS) occurs after ICL implantation, it is crucial to assess whether the ICL size is appropriate besides controlling the IOP. In addition, alternative TICLs often need to be custom-made. During prolonged waiting periods for replacement, provisional ICL realignment will predict simulation for planned exchange. In such cases of mydriasis, minimizing the use of mydriatic drugs is critical to avoid worsening preexisting iris sphincter dysfunction. Pupil constrictors should only be used to assess iris sphincter function and are not recommended as routine therapy for these patients.

## Data Availability

All data and material collected during this study are available from the corresponding author upon reasonable request.
